# Irritational Fibroma Mimicking an Odontogenic Infection: A Case Report of a Misdiagnosed Extraoral Fibroma

**DOI:** 10.7759/cureus.56311

**Published:** 2024-03-17

**Authors:** Bader Fatani, Abdulrahman I Alhilal, Faris A Alghamdi, Nawaf A Alfawaz, Muhannad A Alhaqbani, Fahad S Almutairi, Hesham S AlRfydan

**Affiliations:** 1 Dentistry, College of Dentistry, King Saud University, Riyadh, SAU; 2 Oral and Maxillofacial Surgery, Ministery of Health - Qassim Cluster, Qassim, SAU

**Keywords:** trauma, biopsy, irritational fibroma, oral fibroma, fibroma

## Abstract

Fibroma is a benign fibrous tissue growth that develops in response to injury or irritation. It is usually firm, painless, nodular, and merging in color with the surrounding tissue. Commonly located in areas such as the buccal mucosa, tongue, and lip, the usual treatment involves surgical removal. In this case report, we present a rare instance of misdiagnosed extraoral irritational fibroma that emerged following the surgical extractions of the lower left third molar.

## Introduction

A fibroma is a noncancerous scar-like response commonly triggered by prolonged irritation. It is known by various names such as oral polyp, fibrous nodule, traumatic fibroma, and fibrous hyperplasia. The condition often arises from chronic irritation such as lip or cheek biting, dental trauma, orthodontic treatments, or dental prostheses. The fibroma typically matches the intraoral mucosa lining color, but it can sometimes appear lighter or darker if it is associated with internal bleeding. Trauma may cause the surface to roughen, scale, or develop ulcers. Usually dome-shaped, it resembles a pedunculated polyp, with traumatic fibroma mostly found in the buccal mucosa, gingiva, tongue, and lower lips [1,2]. Fibrous connective tissue benign tumors are often found in the oral cavity, with most being inflammatory rather than neoplastic. True oral mucosa fibromas are very uncommon benign growths [3]. Inflammatory hyperplastic lesions involve tissue enlargement owing to cell increase in response to injury. Traumatic irritants such as calculi, overhanging margins, restorations, and others can trigger these lesions. Fibrous hyperplasia, a type of these lesions, is also known as the healed consequence. They manifest as growths on the mucous membrane and are usually small, with rare larger ones. These lesions are noncancerous, and recurrences are often because of unresolved chronic irritation [4,5]. In this case report, we demonstrate a rare case of misdiagnosed extraoral irritational fibroma that developed following the surgical extraction of the lower-left third molar.

## Case presentation

**Figure 1:**
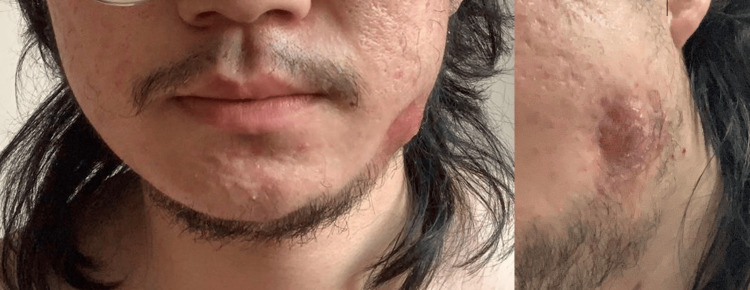
Demonstration of the lower-left extraoral swelling.

This is a case of a 22-year-old male patient with no relevant medical history and no known allergies. The patient presented to the dental emergency clinic complaining of pain and persistent lower-left extraoral hard swelling that started two weeks following the surgical extraction of the lower-left third molar (Figure 1). The swelling was tender to the touch, and the patient experienced on-and-off pressure pain that was not provoked by any stimulus.

**Figure 2:**
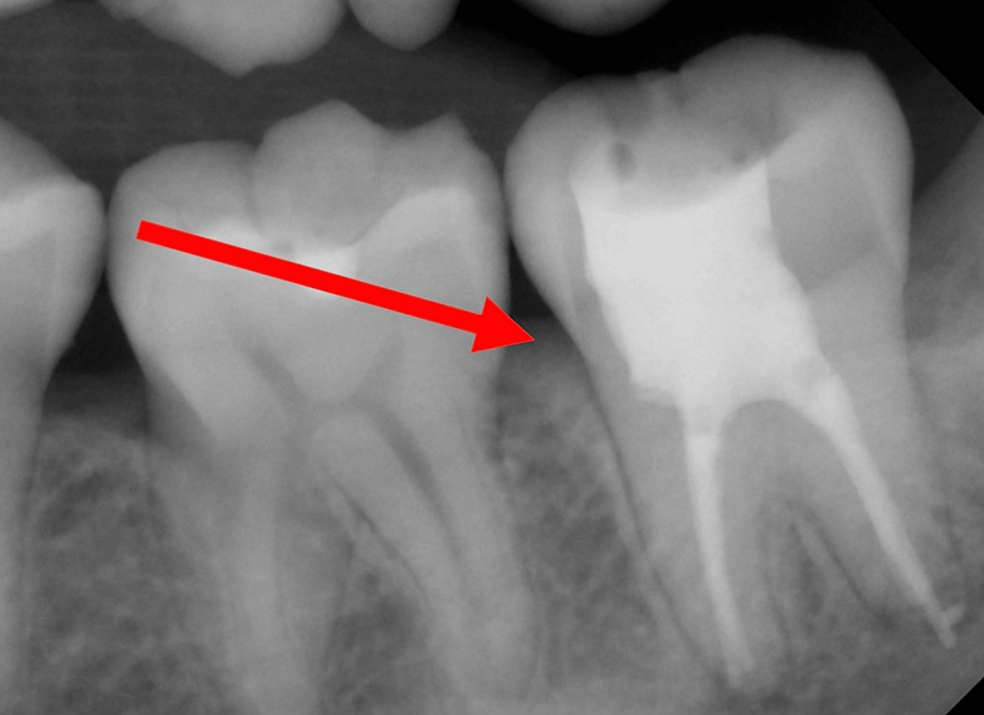
Endodontic treatment of lower-left second molar.

The patient was prescribed amoxicillin with clavulanic acid 1 g two times a day by his previous dentist with no reported improvement. The patient was then seen by the endodontic department, which then misdiagnosed the swelling as of pulpal origin and initiated a full endodontic therapy of the lower-left second molar, followed by an endodontic retreatment two weeks later. Figure 2 shows the endodontic treatment of the lower-left second molar.

**Figure 3:**
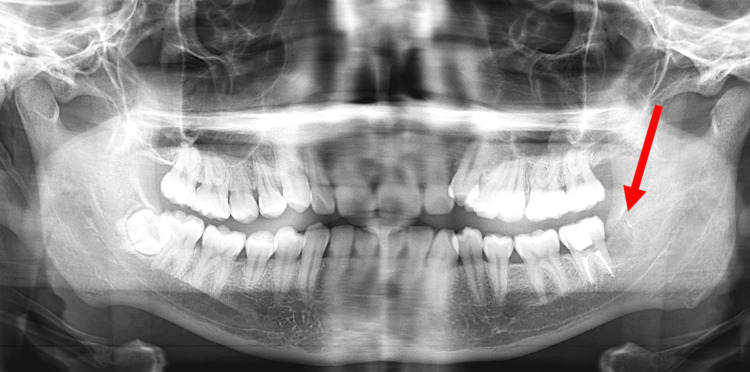
Panoramic radiograph showing the extracted site of the lower-left third molar.

Following endodontic therapy, the patient experienced no improvement in swelling size and no reduction in pain level. The patient was then referred to the oral and maxillofacial surgery clinics for further assessment. A panoramic radiograph was then taken, showing the extracted site of the lower-left third molar and endodontic-treated lower-left second molar in Figure 3.

**Figure 4:**
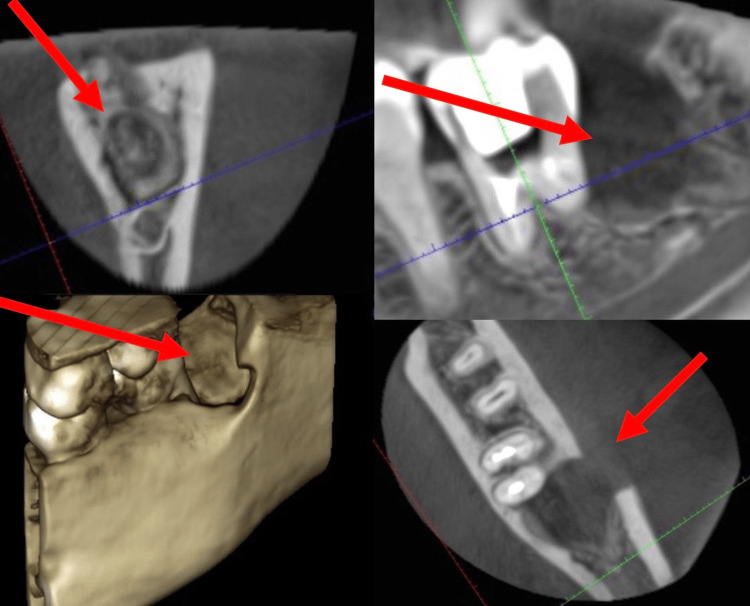
Cone beam computed tomography of the extracted site.

The patient was diagnosed initially with an odontogenic abscess that originated following the surgical extraction of the lower-left third molar, which further required an intraoral incision that showed no pus drainage in the surgical site. Amoxicillin with clavulanic acid 1 g two times a day and metronidazole 500 mg three times a day was then prescribed for 10 days with no further reduction in both swelling size and pain perception. A cone beam computed tomography (CBCT) was then requested. CBCT of the extracted site is presented in Figure 4.

Nonresponsiveness to initial surgical pharmacological and surgical treatment entails the need for a complete excisional biopsy of the lesion under local anesthesia. The patient was then administered 2% lidocaine with epinephrine 1:100,000 as a local infiltration around the lesion. A complete surgical excisional biopsy of the lesion using a surgical blade was made. The biopsy was sent in 10% formalin to the histopathologist. The results demonstrated scar tissue with patchy chronic inflammation, which was further diagnosed as an irritational fibroma.

## Discussion

Identifying common oral conditions enables healthcare providers to diagnose and treat patients in a primary care setting or refer the patient to a suitable healthcare provider such as a dentist, oral surgeon, otolaryngologist, or other relevant specialist [6]. An oral fibroma is considered a harmless growth of fibrous tissue that forms in reaction to trauma or irritation. It is typically firm, nodular, and painless, with a color resembling the surrounding tissue. It is found in areas such as the buccal mucosa, tongue, and lip, and the standard treatment is surgical removal. Fibromas also have a low likelihood of returning [7]. Diwan et al. discussed treating traumatic fibromas using a scalpel and diode laser. The author explained that it is crucial to diagnose, treat the cause, and inform the patient regarding the lesion. The diagnosis is confirmed through clinical features and trigger analysis. Surgical excision is the main treatment option for oral or traumatic fibromas and is widely used [1]. Christopoulos et al. presented a similar case of a 44-year-old Caucasian woman who underwent an examination for growth on the right side of her upper jaw that had been noticed 18 months earlier. Initially suspected to be an alveolar abscess, her dentist performed endodontic treatment on teeth 11, 12, and 13 and prescribed antibiotics [3]. This treatment was not successful. Following an excisional biopsy and analysis of microscopic and immunohistochemical characteristics, a diagnosis of true fibroma was confirmed. Moreover, Rangeeth et al. provided a case of a nine-year-old girl who presented with several lumps on her lower lip, diagnosed as fibroma and mucocele through clinical and histological examination. Surgical removal under local anesthesia was conducted without any further complications during recovery [4]. In a study by Fowler et al. on 31 cases of fibromatosis in the mouth and surrounding areas, patients were aged from birth to 51 years, with 74% occurring in the first 10 years of life [8]. The main symptom was a painless mass in the cheek, tongue, or submandibular region. Bone erosion was common in lesions near the jaw. Following surgical removal, five patients experienced recurrence, with a rate of 23.8%. Histopathologically, irritation fibroma may exhibit an intact or ulcerated stratified squamous epithelium with shortened and flattened rete pegs. Treatment involves removing causative factors, scaling nearby teeth, and completely excising the lesion along with the affected periodontal ligament and periosteum to reduce recurrence risk. Any known irritants such as poorly fitting dental appliances or rough restorations should be eliminated. Long-term follow-up is crucial because of the high likelihood of recurrence if the lesion is not fully removed [9]. Fibroma should be considered when a patient shows a new lesion, especially if it is linked to a recent injury or chronic inflammation. As a fibroma's differential diagnosis includes other benign conditions and rare malignant neoplasms, a biopsy removing the lesion can confirm the diagnosis and offer potential treatment [10].

## Conclusions

This case report demonstrates the case of a rare extraoral irritational fibroma that developed following the extraction of the lower-left third molar. The extraoral swelling did not respond to initial antibiotic or surgical therapy. Following the exclusion of an active infection, the final excisional biopsy was determined, and the result indicated an irritational fibroma. When new abnormal growth develops particularly in association with a recent injury or persistent inflammation, the possibility of an irritational fibroma should be considered. Given that fibromas must be distinguished from similar benign lesions and uncommon cancerous tumors, performing a biopsy to remove and examine the lesion is crucial for confirming the diagnosis and providing a successful means of treatment.

## References

[1] Diwan B, Shirbhate U, Bajaj P, Reche A, Pahade A (2023). Conventional scalpel and diode laser approach for the management of traumatic fibroma. Cureus.

[2] Gonsalves WC, Chi AC, Neville BW (2007). Common oral lesions: part II. Masses and neoplasia. Am Fam Physician.

[3] Christopoulos P, Sklavounou A, Patrikiou A (1994). True fibroma of the oral mucosa: a case report. Int J Oral Maxillofac Surg.

[4] Rangeeth BN, Moses J, Reddy VK (2010). A rare presentation of mucocele and irritation fibroma of the lower lip. Contemp Clin Dent.

[5] Toida M, Murakami T, Kato K (2001). Irritation fibroma of the oral mucosa: a clinicopathological study of 129 lesions in 124 cases. Oral Med Pathol.

[6] Randall DA, Wilson Westmark NL, Neville BW (2022). Common oral lesions. Am Fam Physician.

[7] Valério RA, de Queiroz AM, Romualdo PC, Brentegani LG, de Paula-Silva FW (2013). Mucocele and fibroma: treatment and clinical features for differential diagnosis. Braz Dent J.

[8] Fowler CB, Hartman KS, Brannon RB (1994). Fibromatosis of the oral and paraoral region. Oral Surg Oral Med Oral Pathol.

[9] Lanjekar A, Kulkarni S, Akhade S, Sonule S, Rathod U (2016). An unusually large irritation fibroma associated with gingiva of lower left posterior teeth region. Case Rep Dent.

[10] Cohen PR (2022). Biting fibroma of the lower lip: a case report and literature review on an irritation fibroma occurring at the traumatic site of a tooth bite. Cureus.

